# Student’s Manual of Electro-Therapeutics

**Published:** 1884-10

**Authors:** 


STUDENT'S MANUAL OF ELECTRO-THERAPEUTICS, embodying Lectures delivered in the course of Therapeutics at the Woman's Medical College of the New York Infirmary. By R. W. Amidon, A. M., M. D., Secretary of American Neurologic Association; member of the New York Neurological Society of the New York Academy of Medicine, and of the New York Pathological Society; Lecturer on Therapeutics at the Woman's Medical College of the New York Infirmary, etc. G. P. Putnam's Sons, New York, 27 and 29 West 23d street; London, 25 Hamilton street, Covent Garden. 1884.

This modest title embodies a concise treatise on the principles and practice of Electro-Therapeutics, under the head of Galvanism, Faradism, with reaction of degeneration of nerves, and Electro-diagnosis with the therapeutic applications of electricity to the muscular system, spinal disease, cerebral disease, spasmodic affections, anesthesia, hyperesthesia and chronic rheumatism.

It has the very unusual merit of neither adding to or subtracting from the requisites for understanding the matter treated of, and in these days of book-making, it is a most commendable result to get up so small a volume upon such an important subject, which covers the ground to the entire satisfaction of the searcher after knowledge in this department.

It is a labor-saving process for the student, and is entitled to the careful study of all concerned.

